# Sleep-Related Cognitive Processes and the Incidence of Insomnia Over Time: Does Anxiety and Depression Impact the Relationship?

**DOI:** 10.3389/fpsyg.2021.677538

**Published:** 2021-06-21

**Authors:** Annika Norell-Clarke, Mikael Hagström, Markus Jansson-Fröjmark

**Affiliations:** ^1^Department of Social and Psychological Studies, Karlstad University, Karlstad, Sweden; ^2^Department of Health Sciences, Kristianstad University, Kristianstad, Sweden; ^3^Department of Psychology, Lund University, Lund, Sweden; ^4^Department of Clinical Neuroscience, Centre for Psychiatry Research, Karolinska Institutet, Stockholm, Sweden; ^5^Stockholm Health Care Services, The Centre for Psychotherapy, Education & Research, Region Stockholm, Stockholm, Sweden

**Keywords:** insomnia, incidence, arousal, epidemiology, anxiety, depression, safety behaviors, worry

## Abstract

**Aim:** According to the Cognitive Model of Insomnia, engaging in sleep-related cognitive processes may lead to sleep problems over time. The aim was to examine associations between five sleep-related cognitive processes and the incidence of insomnia, and to investigate if baseline anxiety and depression influence the associations.

**Methods:** Two thousand three hundred and thirty-three participants completed surveys on nighttime and daytime symptoms, depression, anxiety, and cognitive processes at baseline and 6 months after the first assessment. Only those without insomnia at baseline were studied. Participants were categorized as having or not having incident insomnia at the next time point. Baseline anxiety and depression were tested as moderators.

**Results:** Three cognitive processes predicted incident insomnia later on. Specifically, more safety behaviors and somatic arousal at Time 1 increased the risk of developing insomnia. When investigating changes in the cognitive processes over time, reporting an increase of worry and safety behaviors also predicted incident insomnia. Depressive symptoms moderated the association between changes in worry and incident insomnia.

**Conclusion:** These findings provide partial support for the hypothesis that cognitive processes are associated with incident insomnia. In particular, safety behaviors, somatic arousal, and worry increase the risk for incident insomnia. Preventative interventions and future research are discussed.

## Introduction

Insomnia is a common disorder that is characterized by sleep initiation and maintenance difficulties. For a diagnosis to be made, the sleep difficulties must cause distress or an impaired ability to function in important areas, e.g., in social life or in a work context (American Psychiatric Association, 2000, 2013).

Although insomnia is often a persistent or reoccurring disorder over time, there is also evidence that insomnia also has relatively high rates of incidence. Prospective investigations examining the incidence of insomnia have documented that 3–20% of those without insomnia at previous assessments develop insomnia later (Ford and Kamerow, 1989; Katz and McHorney, 1998; Foley et al., 1999; Quan et al., 2005; Jansson and Linton, 2006; LeBlanc et al., 2009). The identification of risk factors for incident insomnia is critical for understanding the etiology of insomnia as well as for preventative interventions.

The so-called “3P model” has been widely used as a conceptual framework to understand the development of insomnia (Spielman, 1986; Spielman and Glovinsky, 1991; Morin, 1993). Specifically, this model suggests that the susceptibility to developing insomnia is increased by a combination of predisposing (e.g., stress reactivity) and precipitating factors (e.g., a stressful live event). The current evidence indicates that factors associated with developing insomnia include socio-demographic parameters, such as gender (Jansson and Linton, 2006; Gureje et al., 2011), a previous episode or family history of insomnia (LeBlanc et al., 2009; Morin et al., 2009), stressful life events or stress reactivity (Healey et al., 1981; Bastien et al., 2004; Jarrin et al., 2014), psychosocial work stressors (Jansson and Linton, 2006), and health-related issues such as pain, anxiety, depression, and disability (Foley et al., 1999; Jansson-Fröjmark and Lindblom, 2008; Kim et al., 2009; LeBlanc et al., 2009; Gureje et al., 2011; Jansson-Fröjmark and Boersma, 2012). Further, once insomnia has developed, the perpetuating factors such as sleep-disturbing habits, negative thinking styles, and misperceptions about sleep become the driving force in persistent insomnia (Harvey, 2002; Perlis et al., 2011). Most insomnia models focus on this category of factors as they explain how insomnia can be treated but a question that is insufficiently answered is whether the perpetuating factors may also be risk factors for incident insomnia.

Amongst the perpetuating factors, cognitive processes have received increased attention in the maintenance of insomnia since the introduction of the Cognitive Model of Insomnia in 2002 (Harvey, 2002), although cognitive factors are included in other prominent insomnia models also (Morin, 1993; Perlis et al., 1997; Lundh and Broman, 2000; Espie, 2002; Buysse et al., 2011). The models share some processes and include worry, unhelpful beliefs about sleep, sleep-disturbing arousal (cognitive and somatic), selective attention and monitoring for threats, and safety behaviors (maladaptive habits). Worry, both in the form of insomnia-specific and general thought processes, is viewed as triggering arousal, increasing nighttime symptoms and to be maintained by unhelpful beliefs and selective attention and monitoring (noticing more reasons to be worried; Gross and Borkovec, 1982; Harvey, 2005; Carney and Waters, 2006). Experimental studies have found worry to be associated with insomnia symptoms, both when measured subjectively (diaries, questionnaires) and objectively through EEG (Galbiati et al., 2017). Unhelpful beliefs about sleep are regarded as triggering worry, influencing the use of safety behaviors to avoid feared outcomes, and being linked to improvements following cognitive behavioral therapy for insomnia (Morin et al., 1993; Harvey, 2005; Woodley and Smith, 2006; Jansson-Fröjmark and Linton, 2008; Schwartz and Carney, 2012). Somatic, cognitive, and cortical arousal is often viewed as conditioned to the bed and bedroom through spending excessive time in bed while awake, thereby increasing nighttime symptoms (Perlis et al., 1997; Drummond et al., 2004; Jansson and Linton, 2007; LeBlanc et al., 2009; Riemann et al., 2010). Arousal is primarily not a cognitive process but has been related to cognitive factors in existing conceptualizations of insomnia and therefore needs further probing when cognitive processes are investigated (Espie, 2002; Harvey, 2002). Selective attention and monitoring of sleep-related threats is viewed as a cognitive process that interrupts the shift between being awake and falling asleep (Espie et al., 2006). Experimental investigations have shown that patients with insomnia are selectively attentive to sleep-related threats (e.g., Spiegelhalder et al., 2009; Woods et al., 2009, 2013). Safety behaviors are viewed as attempts to avoid feared outcomes (e.g., napping to feel more energized and suppressing unwanted thoughts while trying to fall asleep). Safety behaviors are often seen by the patient as helpful but may have undesirable long-term effects and prevent unhelpful beliefs from being tested and corrected (Salkovskis, 1991; Morin, 1993; Ree and Harvey, 2004).

Our previous research, using epidemiological methodology, has shown that sleep-related cognitive processes are associated with insomnia. First, those with insomnia, relative to poor and good sleepers, report more worry, unhelpful beliefs, arousal, selective attention and monitoring, and safety behaviors (Jansson-Fröjmark et al., 2012). Second, through a prospective design, we have also shown that the very same cognitive processes distinguished between those with persistent insomnia and those with normal sleep (Norell-Clarke et al., 2014). For those with insomnia, more worry about sleep at baseline predicted persistent insomnia instead of remission later. A decrease of selective attention and monitoring, and less use of safety behaviors over time predicted remission from insomnia. In general, these results remained when psychiatric symptoms and somatic complaints were added to the models.

Conclusively, the cognitive processes are linked to the persistence and remission of insomnia. However, less is known of the association between cognitive processes and incident insomnia over time. Puzzlingly, as the Cognitive Model of Insomnia suggests that the processes lead to a real sleep deficit over time, only a few published studies have investigated cognitive factors in relation to insomnia incidence. Studies investigating sleep-related beliefs (Jansson-Fröjmark and Linton, 2008), a ruminative style and somatic sensitivity (Gosling et al., 2012), and arousal or arousability (Jansson-Fröjmark and Lindblom, 2008; LeBlanc et al., 2009) found that these factors are associated with an increased risk for incident insomnia. Anxiety and depression are often comorbid with insomnia (e.g., Ford and Kamerow, 1989) and have been found to share a bidirectional relationship with insomnia (e.g., Jansson-Fröjmark and Lindblom, 2008). Although comorbidity with anxiety or depression does not seem to diminish the effects from cognitive behavioral therapy for insomnia (Bélanger et al., 2016), less is known on how these symptomatologies might interact with cognitive processes through the development of insomnia.

The overall aim of this investigation was to expand on the previous research by examining the association between sleep-related cognitive processes from the Cognitive Model of Insomnia and the incidence of insomnia in the general population. The five cognitive processes that were investigated were worry, unhelpful beliefs about sleep, arousal, selective attention and monitoring, and safety behaviors (the sixth factor, distorted perception of deficit, was not indexed since objective sleep measurement was not possible in this epidemiological study). As anxiety and depression have been identified as potential predictors of insomnia (Jansson-Fröjmark and Lindblom, 2008), they were tested as moderators in the analyses. None of the participants fulfilled criteria for insomnia at baseline and the classification of incident insomnia was executed 6 months later. More specifically, two aims were explored.

1. The first aim was to investigate whether the degree of dysfunctional insomnia-related cognitive processes from the Cognitive Model of Insomnia can discriminate between those who go on to develop incident insomnia and those who do not. The hypothesis was that elevated scores on the cognitive processes at baseline would increase the risk of developing incident insomnia later.
2. The second aim was to examine whether those who reported increases in the cognitive processes over time would have a higher risk of developing incident insomnia later on. The hypothesis for the second aim was that a worsening (increase) in the cognitive processes over time would increase the risk of developing incident insomnia later on.

## Materials and Methods

### Overview of the Design

**Figure 1:**
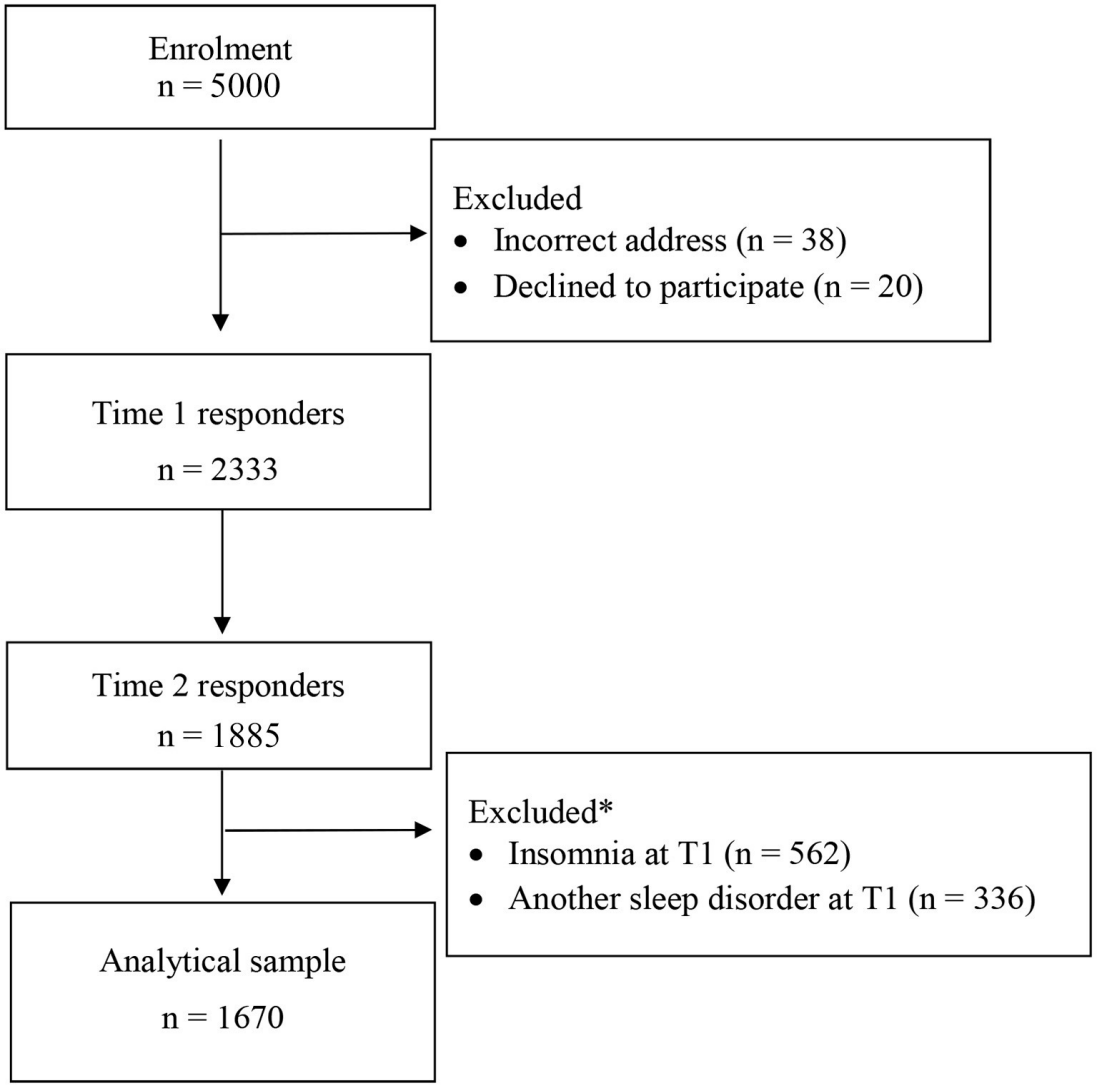
Flow chart of recruitment into the PIPPI study. *Some excluded cases fulfilled both exclusion criteria regarding sleep disorders at Time 1.

This paper uses data from the Prospective Investigation on Psychological Processes for Insomnia (PIPPI) study. The study was approved by the Regional Ethics Board in Uppsala, Sweden. A survey regarding demographic factors, nighttime symptoms, daytime impairment, primary sleep disorders, psychiatric and somatic disorders, and sleep-related cognitive processes was sent to a randomly selected sample from the general population in two Swedish counties at three time-points over 18 months (i.e., September 2008, March 2009, and April 2010). Data from the first and second time points were used in this longitudinal study, as this included the greatest number of participants. See Figure 1 for a flow chart. All data were collected by postal surveys. When participants did not respond to the initial survey or a reminder after 2 weeks, this was viewed as declination to participate. Those who responded at time 1 were sent a postal survey at time 2 and at time 3. To improve the response rates at the three time-points, several procedures were adopted from a Cochrane review (Edwards et al., 2007).

### Participants

A random sample of 5,000 participants (age range 18–70 years) was the target group. The sample was procured from the national register (a listing of all Swedish residents). The randomization was organized by the national statistics department in Sweden (Statistics Sweden). Of the random sample, 2,333 individuals (47.1% of those that were eligible) returned the survey at time 1, and 1,887 at time 2. On average, the participants were 47.9 years, and the majority were women (56.6%). Related to marital status, the majority were cohabitant or married or having a partner (80.1%), while 12.85 reported being single, 4.0% being divorced, and 1.6% being widowed. As for vocational status, most participants reported being employed or students (73.1%), and the remaining individuals were unemployed, on sick leave, on pension or other status. As for educational level, the majority had completed high school (43.3%), while 29.7% had finished college or university and 25.4% compulsory school. Most of the participants, 91.3%, were born in Sweden. Comparisons with register data showed that our sample was representative for the Swedish population regarding age, gender, relationship status, occupational status, educational level, and reported sleep disturbance (Jansson-Fröjmark et al., 2012). Attrition analyses showed no differences for gender, sleep disturbance, or insomnia severity although non-responders were more likely to be younger than responders. The attrition analyses have been described in detail elsewhere (Jansson-Fröjmark et al., 2012).

The criteria for the current study were two-fold: (1) that the participants did not fulfill criteria for insomnia at baseline (*n* = 1,771), and (2) that the participants did not meet criteria for another primary sleep disorder than insomnia (*n* = 1,997). Of the 2,333 baseline responders, 1,670 fulfilled both abovementioned criteria for inclusion. The socio-demographic characteristics for the 1,670 qualifying study participants are displayed in Table 1. As can be seen in the table, the mean age was approximately 50 years, and there was a slight overrepresentation of women in the sample (52%). More than 80% were cohabitant, married or having a partner, and approximately 75% were employed or students. More than 70% had finished compulsory school or high school, and the majority of the participants (93.3%) were born in Sweden.

**Table 1:** Study participants (*N* = 1,670): descriptive statistics.

		***M* (*SD*) or %**
Age (mean)		47.9 (14.5)
Gender (female)		52.0%
Marital status	Cohabitant, married or having a partner	81.4%
	Single, divorced or widowed	18.6%
Vocational status	Employed or student	75.1%
	Unemployed, sick leave, pension or other status	24.9%
Educational level	Compulsory school or high school	70.9%
	College or university	29.1%
Ethnicity (born in Sweden)		93.3%

### Self-Report Instruments

#### Nighttime Symptoms

To assess nighttime symptoms, the participants were asked to complete the following categorical questions based on the past month (Edinger et al., 2004): sleep onset latency (SOL; <15 min, 16–30 min, 31–60 min, >60 min), wake time after sleep onset (WASO; same alternatives as for SOL), early morning awakening (EMA; same alternatives as for SOL). To determine sleep disturbance, the participants were asked to complete the following question based on the past month: “Would you say that you have had trouble sleeping? (yes or no).”

#### Daytime Symptoms

The participants were instructed to report on the degree of the following sleep-related daytime symptoms during the past month (Edinger et al., 2004): fatigue/malaise, impairment in attention, concentration, or memory, social dysfunction, vocational dysfunction, mood disturbance, irritability, daytime sleepiness, reduction in motivation, energy, or initiative, proneness for errors or accidents at work or while driving, tension headaches, gastrointestinal symptoms, and concerns or worries about sleep. The response alternatives for these indications of daytime symptoms were: not at all (1), somewhat (2), quite a bit (3), and a lot (4). The response alternatives for the three functional domains were: not affected (1), some difficulties but not less ability to function (2), less ability to function (3), much less ability to function (4) could barely function (5).

#### Sleep Disorders

The SLEEP-50 was employed to determine five DSM-IV-TR sleep disorders: apnea, narcolepsy, restless legs/periodic limb movement disorder, circadian rhythm disorder, and sleep walking (Spoormaker et al., 2005). This measure has displayed acceptable psychometric properties, e.g., high internal consistency, satisfactory test–retest reliability (0.71–0.88), and a factor structure that is in concordance with the DSM system (American Psychiatric Association, 2000). Further, the instrument has demonstrated reasonable sensitivity and specificity scores, and acceptable agreement between clinical diagnoses and classification derived from the SLEEP-50 (kappa = 0.77). The response alternatives were from 1 (not at all) to 4 (very). In the current sample, the internal consistency was 0.92.

#### Sleep-Related Cognitive Processes

The Anxiety and Preoccupation about Sleep Questionnaire (APSQ) was used to assess sleep-related worry (Tang and Harvey, 2004). The response alternatives on the 10 items were changed slightly to make the alternatives similar to the other psychological process measures in the survey (1–5; 1 = strongly disagree, 5 = strongly agree). The score range was thus 10–50. The version with revised response alternatives has displayed acceptable psychometric properties (Jansson-Fröjmark et al., 2011). For the current sample, the internal consistency was 0.93.

The Dysfunctional Beliefs and Attitudes about Sleep Scale (DBAS) was used to capture dysfunctional beliefs (Morin et al., 1993). A condensed version with 10 items (the DBAS-10) was used to identify sleep-related dysfunctional beliefs (Espie et al., 2000). To match the other measures' response alternatives, the alternatives were changed (1–5; 1 = strongly disagree, 5 = strongly agree), and the score range was thus 10–50. In the current sample, the internal consistency was 0.85.

The somatic subscale from the Pre-Sleep Arousal Scale was employed to determine physiologic arousal (PSAS-S; Nicassio et al., 1985). The subscale consists of eight items. The response alternatives for the items were 1 (not at all) to 5 (extremely). The score range was 8–40. Based on data from the PIPPI project (Jansson-Fröjmark and Norell-Clarke, 2012), the Cronbach's alpha of the PSAS-S was acceptable at 0.72.

Monitoring and attentional bias regarding sleep was measured with the Sleep Associated Monitoring Index (SAMI). The current study consisted of eight combined items, which were theoretically derived from the original 30 items. Each of the eight items represented a subscale each. As the eight subscales are highly correlated with the subscale totals (*r*: 0.71–0.94; Neitzert Semler and Harvey, 2007), this seemed reasonable. There is a high correlation (0.86) between the 30-item and the 8-item SAMI versions (Jansson-Fröjmark and Sunnhed, 2020). The response alternatives ranged from 1 (not at all) to 5 (all the time). The score range for the 8-item scale was 8–40. Based on the current sample, the Cronbach's alpha for the total scale was acceptable at 0.81 (Kline, 1993).

Sleep-related safety behaviors were measured with the Sleep-Related Behaviors Questionnaire (SRBQ; Ree and Harvey, 2004). When reducing the scale from 32 to 16 questions, the remaining items were chosen for their higher correlations with the Insomnia Severity Index (ISI; correlations from Ree and Harvey, 2004) in a sample of insomnia patients, and healthy subjects from the general population and university students. In an unpublished pilot study, it was shown that there was a high correlation (0.92) between the 32-item and the 16-item SRBQ versions. The response alternatives were 0 (almost never)−4 (almost always) (score range 0–64). Based on the current sample, the internal consistency was high (0.88) for the shortened 16-items SRBQ scale. The correlations between the cognitive processes are available in Table 2.

**Table 2:** Correlations between the five self-report measures assessing insomnia-related cognitive processes at Time 1 (*N* = 1,670).

	**APSQ**	**DBAS**	**PSAS-S**	**SAMI**
APSQ				
DBAS	0.63^**^			
PSAS-S	0.45^**^	0.37^**^		
SAMI	0.57^**^	0.52^**^	0.55^**^	
SRBQ	0.66^**^	0.52^**^	0.48^**^	0.60^**^

*Pearson's correlation coefficient was used as correlation statistics. APSQ, anxiety and preoccupation about sleep questionnaire; PSAS-C, pre-sleep arousal scale-cognitive subscale; DBAS, dysfunctional beliefs and attitudes about sleep*.

** *p < 0.01*.

### Covariates

The Hospital Anxiety and Depression Scale was used to assess anxiety and depression (HADS; Zigmond and Snaith, 1983). The HADS is a self-rating scale with 14 questions in which the severity of anxiety (HADS-A) and depression (HADS-D) is rated on 4-point scales (score range 0–21). Based on the current sample, the internal consistency for the total scale was high at 0.90 (anxiety: 0.85, depression: 0.84). To detect a possible case of anxiety or depression, an often-used procedure in research has been to dichotomize the two subscales: a score of 7 or less indicates a non-case and a score of 8 or more indicates a case. This cut-off was determined based on a review in which the above cut-offs resulted in an optimal balance between sensitivity and specificity in comparison to diagnostical interviews (Bjelland et al., 2002). Although a recent review has reported that the factor structure of the HADS varies between one to five in studies (Cosco et al., 2012), it should be noted that a majority of the included psychometric studies found support for a two factor solution, and that studies using Swedish samples have consistently found support for two distinct factors (e.g., Lisspers et al., 1997; Hansson et al., 2009; Djukanovic et al., 2017).

### Insomnia Classification

The participants were classified into groups according to their sleep patterns, daytime impairment, and evidence of sleep disorders other than insomnia. The classification used an algorithm based on a combination of insomnia diagnostic criteria from Research Diagnostic Criteria for insomnia (Edinger et al., 2004), established quantitative criteria for insomnia (Lichstein et al., 2003), and screening for sleep disorders other than insomnia (Spoormaker et al., 2005). To fulfill criteria for insomnia, four diagnostic criteria needed to be met: (1) The participant affirmed a sleep disturbance during the past month. (2) The individual reported initial, middle, or late insomnia (31 min or more per night awake involuntarily at any stage during an estimated average night; Lichstein et al., 2003). (3) The participant reported daytime impairment (a score of 3–5 on at least one item assessing daytime symptoms or impaired function distress). (4) The individual did not meet the criteria for apnea, narcolepsy, restless legs syndrome/periodic limb movement disorder, circadian rhythm disorder, or sleepwalking as assessed with the SLEEP-50. At baseline, none of the study participants fulfilled the criteria for insomnia. At follow-up, the above mentioned criteria were used to categorize the participants into those who had developed insomnia (incident insomnia) and those who had not.

Based on the current sample, the agreement between the current insomnia definition and two established ISI cutoffs were examined (Morin et al., 2011). The ISI measures subjective insomnia severity and has widely been used as a measure of change after treatment. Eight points reflect subthreshold insomnia and 10 points insomnia. The cutoffs displayed very high classification rates (98%) in differentiating those with insomnia compared with controls. The agreement between the ISI cutoffs and the current study's definition was acceptable: the cutoff at eight points correctly classified 99.4% of participants with insomnia and the higher cutoff at 10 points 89.4% (Jansson-Fröjmark et al., 2012). The high agreement indicates that the current study's definition is a satisfactory operationalization of insomnia.

### Statistical Analysis

Binary logistic regression was used to explore both hypotheses. Only those without insomnia at baseline were prospectively followed in this study. For the first hypothesis, the predictors consisted of the five cognitive processes at baseline, with incident insomnia (no – yes) at the 6-month follow-up as the outcome. For the second hypothesis, the standardized residual change scores of the five cognitive processes between two time points (i.e., T1 to T2) were predictor variables and incident insomnia (no – yes) was the outcome. The standardized residual is a formula to convey the time 2 score as larger or smaller than the score predicted linearly by the score at time 1 (Cronbach and Furby, 1970). Standardized residual changes scores were more useful than raw change scores since the former take the time 1 score into account, while controlling for possible random errors of measurement (Steketee and Chambless, 1992). The standardized residuals were computed by converting the raw scores to *Z* scores and were further computed as follows: *Z*_2_ – (*Z*_1_ × *r*_12_) in which *Z*_2_ is the follow-up score, *Z*_1_ the baseline score, and *r*_12_ the correlation between both ratings.

Statistical analyses were performed following the guidelines of Hayes (2017) for evaluating predictors and moderators. To analyze proposed baseline variables as potential predictors and moderators, we used the PROCESS macro in SPSS (Hayes, 2017). In these analyses, the significant cognitive process variables were entered as independent variables, incident insomnia (no – yes) at T2 as the dependent variable, and anxiety and depressive symptoms (HADS-A and HADS-D) as the moderator variables. Continuous variables were kept uncategorized in line with guidelines to maximize power and avoid arbitrary dichotomizations of baseline characteristics and misleading results (Hayes, 2017). When evidence was provided for anxiety and depressive symptoms as moderators (i.e., a significant interaction), the interaction effect was probed by constructed regions of significance plots with simple slopes using the Johnson–Neyman technique.

## Results

### Do Sleep-Related Cognitive Processes at Baseline Increase the Risk for Incident Insomnia?

The descriptive statistics for the cognitive processes at baseline across the two groups (incident insomnia: no – yes) and results from logistic regressions are displayed at Table 3. A multivariate logistic regression analysis, entering the five cognitive processes simultaneously as predictor variables, was used to explore whether the cognitive processes at baseline were associated with an increased the risk for incident insomnia. The overall model was significant (*p* < 0.001). Multi collinearity was controlled for but this was not an issue. Two of the processes were related to an increased risk for incident insomnia: pre-sleep somatic arousal and safety behaviors. This means that an elevated degree of pre-sleep somatic arousal and safety behaviors at baseline increased the risk for incident insomnia. Every point on the PSAS and the SRBQ indicated 8% cumulatively higher odds of insomnia at Time 2.

**Table 3:** Cognitive processes at baseline between those with and without incident insomnia at the subsequent assessment: descriptive statistics and multivariate logistic regression analyses.

	***M* (*SD*) or %**	**OR**	**95% CI**
Not insomnia at T1 (*n* = 1,670): Insomnia at T2 (I; *n* = 82) – Not insomnia at T2 (NI; *n* = 1,588)			
APSQ (T1)	NI: 12.2 (4.3)	1.00	0.95–1.06
	I: 15.3 (7.3)		
DBAS (T1)	NI: 18.8 (5.9)	1.01	0.96–1.06
	I: 21.7 (6.8)		
PSAS-S (T1)	NI: 10.5 (2.9)	1.08	1.01–1.17^*^
	I: 12.8 (4.5)		
SAMI (T1)	NI: 14.5 (4.7)	1.01	0.95–1.07
	I: 17.4 (5.3)		
SRBQ (T1)	NI: 6.7 (6.3)	1.08	1.03–1.12^**^
	I: 12.4 (8.3)		

*CI, confidence interval; I, insomnia; M, mean; NI, not insomnia; OR, odds ratio; SD, standard deviation*.

* *Significant at the 0.05 level*.

** *Significant at the 0.01 level*.

In the next step, it was examined whether anxiety and depressive symptoms (HADS-A and HADS-D) moderated the association between the significant predictors above (i.e., pre-sleep somatic arousal and safety behaviors) and incident insomnia. As is displayed in Table 4, anxiety and depressive symptoms did not act as moderators for the association between pre-sleep somatic arousal and incident insomnia, but depressive symptoms emerged as a significant predictor in combination with pre-sleep somatic arousal and safety behaviors, respectively.

**Table 4:** Results from the moderation models examining the effects of two cognitive processes (PSAS-S and SRBQ) and anxiety and depressive symptoms (HADS-A and HADS-D) at baseline on the incidence of insomnia.

	**Model parameters**	**Coefficients**	***SE***	***p***
**PSAS-S (T1) TO INCIDENT INSOMNIA (T2)**				
HADS-A	PSAS-S	0.165	0.043	<0.001
	Predictor: HADS-A	0.072	0.054	0.183
	Interaction: PSAS-S × HADS-A	−0.009	0.007	0.197
HADS-D	PSAS-S	0.154	0.039	<0.001
	Predictor: HADS-D	0.169	0.046	<0.001
	Interaction: PSAS-S × HADS-D	−0.013	0.009	0.134
**SRBQ (T1) TO INCIDENT INSOMNIA (T2)**				
HADS-A	SRBQ	0.103	0.018	<0.001
	Predictor: HADS-A	0.098	0.054	0.071
	Interaction: SRBQ × HADS-A	−0.008	0.005	0.072
HADS-D	SRBQ	0.087	0.018	<0.001
	Predictor: HADS-D	0.142	0.051	0.005
	Interaction: SRBQ × HADS-D	−0.005	0.005	0.328

### Do Changes in Sleep-Related Cognitive Processes Over Time Increase the Risk for Incident Insomnia?

The descriptive statistics for changes in the cognitive processes over time (i.e., T1 to T2) across the two groups (incident insomnia: no – yes) and results from the logistic regressions can be seen in Table 5. A multivariate logistic regression analysis was employed to investigate whether the changes in the cognitive processes from T1 to T2 were associated with an increased the risk for incident insomnia. The overall model was significant. Changes in two of the processes were associated with an elevated risk for incident insomnia: increases in worry and in safety behaviors. This indicates that worsened (increased) worry and safety behaviors over time increased the risk for incident insomnia. Every increased point on the APSQ and the SRBQ meant 10 and 7% higher odds of insomnia at Time 2.

**Table 5:** Changes in cognitive processes over time between those with and without incident insomnia at the subsequent assessment: descriptive statistics and multivariate logistic regression analyses.

	***M* (*SD*) or %**	**OR**	**95% CI**
Not insomnia at T1 (*n* = 1,670): Insomnia at T2 (I; *n* = 82) – Not insomnia at T2 (NI; *n* = 1,588)			
APSQ (T1–T2)	NI: −0.05 (0.69)	1.10	1.03–1.16^*^
	I: 0.85 (1.30)		
DBAS (T1–T2)	NI: −0.04 (0.69)	1.05	0.99–1.11
	I: 0.56 (0.93)		
PSAS-S (T1–T2)	NI: −0.04 (0.67)	1.05	0.96–1.14
	I: 0.61 (1.03)		
SAMI (T1–T2)	NI: −0.03 (0.68)	1.08	0.99–1.16
	I: 0.58 (0.88)		
SRBQ (T1–T2)	NI: −0.07 (0.73)	1.07	1.03–1.12^*^
	I: 0.82 (0.98)		

*CI, confidence interval; I, insomnia; M, mean; NI, not insomnia; OR, odds ratio; SD, standard deviation*.

* *Significant at the 0.01 level*.

As a subsequent step, it was examined whether anxiety and depressive symptoms moderated the association between the significant predictors above (i.e., changes in worry and safety behaviors) and incident insomnia (see Table 6). Depressive symptoms, but not anxiety symptoms, acted as a significant moderator between changes in worry and incident insomnia. Using the Johnson-Neyman technique for identifying the strength and level of significance at levels of the moderator, the significance region for this moderating effect was for depressive symptoms up to 8.58 points on the HADS-D. This suggests that when HADS-D scores were at 8.58 points or lower among the study participants, depressive symptoms moderated the association between changes in worry and incident insomnia. More specifically, increases in worry were associated with an amplified risk for incident insomnia when the HADS-D score was at 8.58 points or lower. However, it is worth noting that only 38 individuals in the dataset scored above 8.58 points on the HADS-D, making the region of significance uncertain (Hayes, 2017). Regarding anxiety symptoms, they emerged as a significant predictor in combination with increased worry (but not with safety behaviors), and depressive symptoms was a significant predictor together with increased worry and safety behaviors, respectively.

**Table 6:** Results from the moderation models examining the effects of changes in two cognitive processes (APSQ and SRBQ) from baseline to 6-months follow-up and anxiety and depressive symptoms (HADS-A and HADS-D) at baseline on the incidence of insomnia.

	**Model parameters**	**Coefficients**	***SE***	***p***
**APSQ (T1-T2) TO INCIDENT INSOMNIA (T2)**				
HADS-A	APSQ	0.193	0.029	<0.001
	Predictor: HADS-A	0.104	0.051	0.040
	Interaction: APSQ × HADS-A	−0.008	0.007	0.239
HADS-D	APSQ	0.211	0.029	<0.001
	Predictor: HADS-D	0.199	0.042	<0.001
	Interaction: APSQ × HADS-D	−0.018	0.007	0.008
**SRBQ (T1-T2) TO INCIDENT INSOMNIA (T2)**				
HADS-A	SRBQ	0.131	0.021	<0.001
	Predictor: HADS-A	0.083	0.054	0.122
	Interaction: SRBQ × HADS-A	−0.001	0.005	0.837
HADS-D	SRBQ	0.140	0.021	<0.001
	Predictor: HADS-D	0.183	0.048	<0.001
	Interaction: SRBQ × HADS-D	−0.008	0.005	0.111

## Discussion

The overall aim of this investigation was to examine the association between sleep-related cognitive processes and the incidence of insomnia. The first aim was to explore if sleep-related worry, unhelpful beliefs about sleep, pre-sleep arousal, selective attention and monitoring, and safety behaviors could discriminate between those who developed incident insomnia and those who did not over time. Our first hypothesis was partly confirmed as a higher degree of safety behaviors and somatic arousal were significant risk factors for incident insomnia 6 months later. The results suggest that an elevated degree of safety behaviors and heightened pre-sleep arousal not only maintain insomnia but also are risk factors for developing incident insomnia. Safety behaviors have, theoretically and empirically, been found to be related to a multitude of psychiatric and somatic health complaints, such as social phobia, health anxiety, pain, and delusions (e.g., Rachman et al., 2008). However, very little is known regarding how safety behaviors are related to the incidence of psychopathology, although research on the development from incident (acute) pain to persistent pain may be relevant to the development of insomnia. Regarding pain, those who are at high risk of developing persistent pain after an injury endorse a higher degree of unhelpful beliefs (fear-avoidance) regarding which behaviors are safe or unsafe when in pain (Westman et al., 2008). Safety behaviors, such as avoiding pain-inducing movements, taking painkillers routinely for longer than prescribed and doing movements in certain (perceived safer) ways maintain chronic pain by interfering with the otherwise intuitive testing of what the body can or cannot do during recovery, and continually challenging oneself to reclaim the abilities that acute pain interfered with (Craske et al., 2014). As sleep-related safety behaviors often involve practices that interfere with the circadian rhythm and sleep homeostasis, such as napping, spending an excessive amount of time in bed, and affecting one's wakefulness through caffeine, alcohol or nicotine, it is reasonable that they could cause sleep problems over time. As the pain-related safety behaviors are activated only after acute pain, it would be interesting to know if people with a high degree of sleep-related safety behaviors have experienced acute insomnia, and developed new strategies as a result. That somatic arousal functioned as a risk factor for insomnia is in line with previous studies of incident insomnia (Jansson-Fröjmark and Linton, 2008; LeBlanc et al., 2009) and also in a multitude of investigations in those with persistent insomnia (Riemann et al., 2010).

The second aim was to investigate whether increases in sleep-related cognitive processes over time would increase the risk of developing incident insomnia later on. Again, the hypothesis was partly confirmed as increases in worry and safety behaviors were risk factors. These findings thus indicate that reporting more worry and safety behaviors over time constituted a distinct risk for developing incident insomnia. Analyses of changes in risk factors for incident insomnia are almost non-existent (for an exception see LeBlanc et al., 2009) despite that fluctuations in risk factors over time are to be expected and might also inform theory and clinical practice about what drives incident insomnia, apart from baseline scores.

Several theories propose that sleep-related cognitive processes are implicated as maintaining persistent insomnia (Morin, 1993; Perlis et al., 1997; Lundh and Broman, 2000; Espie, 2002; Harvey, 2002; Buysse et al., 2011). Though the cognitive processes appear to be more closely related to the persistence of insomnia in our previous studies (Jansson-Fröjmark et al., 2012; Norell-Clarke et al., 2014), in terms of variance explained, it is important to emphasize that cognitive processes (particularly safety behaviors) were also associated with incident insomnia in this study. This implies that theories with a focus on processes that maintain persistent insomnia might also have heuristic value for understanding the development of insomnia. Combined with the growing evidence for the cognitive model of insomnia (Kaplan et al., 2009; Hiller et al., 2015), the findings from the current study disproves claims that the processes are entirely a secondary effect of the insomnia experience.

Some other findings should be mentioned. Our findings that anxiety and depression functioned as predictor of incident insomnia is in line with previous research (e.g., Jansson-Fröjmark and Lindblom, 2008). The finding that depression moderated the association between increased sleep-related worry and insomnia should be interpreted with caution due to the few participants involved. However, negative thinking, e.g., worry and rumination, can be a symptom of depression (Djukanovic et al., 2017) so it is plausible that depression would amplify the association between increased worry and sleep problems.

A particularly surprising finding was that selective attention and monitoring was not predictive of incident insomnia in multivariate analyses. This was unexpected as several theories view attending to and monitoring of sleep-related threats as maintaining persistent insomnia and later experimental work has found support for attentional bias in persistent insomnia (e.g., Spiegelhalder et al., 2009; Woods et al., 2009, 2013). Also, previous analyses, using the same population, found that a decrease in selective attention and monitoring increased the likelihood of remission from insomnia rather than persistent insomnia, thereby supporting the maintaining role of biased attentional processes (Norell-Clarke et al., 2014). Selective attention and monitoring may therefore be a process that increases its impact over time, with regard to insomnia. The non-significant role for selective attention and monitoring might also be due to the relatively high correlation between the SAMI and the SRBQ (*r* = 0.57). It was also surprising that baseline worry was not predictive of incident insomnia since excessive cognitive activity has been conceptualized as the entry point for persistent insomnia (Harvey, 2002) and because our previous study showed that worry clearly predicted persistent insomnia (Norell-Clarke et al., 2014). Again, the non-significant finding for worry at baseline as a risk factor might be explained by a relatively high association between the APSQ and the SRBQ (*r* = 0.60). Another explanation could be that elevated levels of selective attention or worry may be not enough to trigger incident insomnia: certain overt and covert behaviors (i.e., safety behaviors) motivated by concern for sleep must perhaps develop first for a vicious cycle of insomnia to start spinning.

The current results have implications for screening and preventative interventions. Since validated scales are available to capture sleep-related cognitive processes, screening might benefit from including an assessment of pre-sleep arousal and safety behaviors in particular, as every point indicates an 8% increased risk of insomnia later on. As more recent insomnia research into the role of repetitive negative thought have found support for different associations between worry and rumination, respectively, with subjective and objective measures sleep parameters (Galbiati et al., 2017) rumination should also be investigated. A potential future benefit of focusing on insomnia-related cognitive processes is also that research has shown that it is possible to reverse such factors with targeted interventions. Though this has to date only been demonstrated in patients with persistent insomnia, pilot studies have shown that cognitive therapy for insomnia has been associated with a reduction of worry, unhelpful beliefs, somatic arousal, selective attention and monitoring, and safety behaviors after treatment (Harvey et al., 2007, 2014; Sunnhed et al., 2020). Regarding pre-sleep arousal, which predicted insomnia, relaxation techniques have been associated with beneficial effects on a number of clinical issues, including insomnia, depression, and anxiety (Jorm et al., 2008; Kim and Kim, 2018; Edinger et al., 2021). Whether patients with anxiety and depression might benefit from relaxation as a prevention intervention regarding insomnia, as a way of killing three birds with one stone, is an unanswered question. Also, CBT for insomnia has consistently been associated with reductions of pre-sleep arousal (for a review see Schwartz and Carney, 2012). Future investigations should investigate these and other treatments further in randomized controlled studies to examine whether it is possible to reduce the risk of incident insomnia, particularly among those with subclinical insomnia or in those with insomnia in remission.

### Strengths and Weaknesses

There are a number of methodological strengths with the current investigation. The insomnia-related cognitive processes examined in the current study are underscored in several conceptualizations of insomnia but have never been explored together prospectively before in relation to incident insomnia. Another strength with the study is that other sleep disorders were assessed, although only on the basis of self-report measures. Nevertheless, the current investigation has a number of methodological weaknesses. The moderate response rate (47.1% at baseline) is problematic, as the attrition analysis showed that responders were older than non-responders. Though the response rate might have influenced the results, several studies have showed that high non-response rate does not necessarily have a significant impact on the results when examining associations between variables rather than estimates of a single population parameter (Curtin et al., 2000; Keeter et al., 2006). It should be emphasized that the current sample was almost identical to the Swedish population on several parameters. Another methodological issue is that a proxy for the insomnia construct was used. As a result, it is unknown whether the people classified with insomnia would be diagnosed as such in the health care system. On the other hand, the high agreement between the current study's insomnia definition and validated ISI cutoffs supports the validity of the chosen definition in this study. (Jansson-Fröjmark et al., 2012). Also, it is unknown whether incident insomnia at time 2 captured one or several episodes across time. It is also not established to what degree the participants classified as having insomnia resemble those that seek consultation in the health care system. Based on previous research, it is reasonable to assume that many individuals with sleep difficulties never seek help (Ancoli-Israel and Roth, 1999; Sandlund et al., 2016) and the nature of their problems is therefore largely unknown but it should be noted that those who seek help do so because they experience daytime suffering such as fatigue or mental problems (Sandlund et al., 2016), which the insomnia proxy in the current study included.

Lastly, the longitudinal design cannot confirm a causal relationship between the cognitive processes and incident insomnia. There are potential third factors that could influence the cognitive processes and insomnia, and the processes might also be triggered by insomnia. Future research might examine how the cognitive processes and incident insomnia are inter-related through experimental and preventative designs.

### Conclusions

Because a large group of individuals will develop insomnia over time, it is essential to understand the processes that drive incident insomnia. The current study's results suggested that high degrees of safety behaviors and somatic arousal increase the risk for incident insomnia, and that increases in worry and safety behaviors are associated with higher likelihood of incident insomnia. This creates interesting openings for further research into screening and preventative interventions.

## Data Availability Statement

The data analyzed in this study is subject to the following licenses/restrictions: An anonymized version of data can be made available in response to requests that comply with the ethical principles of good research and that are in keeping with the promises made to participants on the informed consent form. Requests to access these datasets should be directed to markus.jansson-frojmark@ki.se.

## Ethics Statement

The studies involving human participants were reviewed and approved by Regional Ethics Board in Uppsala, Sweden. The patients/participants provided their written informed consent to participate in this study.

## Author Contributions

MJ-F was part of the group that designed the prospective study. AN-C and MJ-F conducted the original data gathering and conceived the research questions for the current manuscript. All authors discussed the statistical approaches prior to the analysis which MJ-F conducted, wrote the first draft of the manuscript, critically reviewed the manuscript, and approved the final version.

## Conflict of Interest

The authors declare that the research was conducted in the absence of any commercial or financial relationships that could be construed as a potential conflict of interest.
